# Editorial: New Insights Into Seed Metabolites: From Research to Application

**DOI:** 10.3389/fpls.2021.726800

**Published:** 2021-08-06

**Authors:** Alma Balestrazzi, Mingxun Chen, Cecilia Silva-Sanchez

**Affiliations:** ^1^Department of Biology and Biotechnology “Lazzaro Spallanzani”, University of Pavia, Pavia, Italy; ^2^Northwest A&F University, Yangling, China; ^3^National Council for Air and Stream Improvement, Inc (NCASI), Cary, NC, United States

**Keywords:** seed metabolites, specialized metabolism, analytical methods, plant breeding, signal molecules, secondary metabolism

Seed metabolites play key roles as a source of nutrients for humans and livestock, or as a supply of raw materials for industries. Besides this, the seed benefits from a wide range of molecules that are timely produced and utilized to promote the transition from the quiescent state of the dry seed to active germination, and later on to facilitate post-germinative growth and seedling establishment. Such a complex developmental strategy requires sophisticated control mechanisms carried out by specific metabolites acting as signal molecules. The latter include carbohydrates (e.g., sucrose and trehalose metabolism) and lipids (including oxylipins, phosphoinositides, sphingolipids). The seed ability to withstand abiotic and biotic stresses is also related to distinctive metabolic profiles (e.g., antioxidant polyphenols) resulting from the integration of exogenous signals with endogenous signaling pathways, finally triggering protective responses. The recent advances in this field of plant science have contributed to disclose several important features of the seed metabolome, highlighting the specific role of different classes of compounds in seed quality and health. To date, there is still a consistent gap of knowledge on these issues that will be possibly overcome through the in-depth, multilevel investigation of seed physiology and biochemistry. All the efforts devoted to elucidate the genetic and molecular bases of seed quality traits will pave the way to powerful applications in crop breeding and seed technologies. This Research Topic provides the latest new insights on the contribution of seed metabolites and related physiological and molecular processes to seed and seedling resilience.

The review by Watt et al. provides an exhaustive up-date on transcription factors that modulate genes responsible for multiple traits, with focus on abiotic stress tolerance and grain size. This is a promising research field that holds a strong potential significance for cereal crop improvement, in view of the current and future climate challenges. The review underlines critical issues, e.g., species-specific components and synteny, and the need to better understand the way microRNAs regulate transcription factors. Seed size issues can be afforded from different perspectives. The work by Curtidor et al. aims at understanding how carbon composition changes during embryo development, contributing to final seed or embryo size. The study was performed using *Arabidopsis thaliana* accessions with medium and large seed sizes. Based on metabolic profiles, the “torpedo-to-mature embryo” and “mature embryo-to-desiccation stage” transitions disclosed distinct carbon markers. Such changes were pronounced in the accession with the large seed size. Also the study (Liu et al.) reports on issues of seed size, looking in this case at the molecular pathways linked to cytokinin action in the model plant *A. thaliana*. The subject of this investigation is the multifunctional *AtENO2* gene encoding the enolase enzyme and AtMBP-1 (c-Myc binding protein 1-like). A mutation in *AtENO2* results into decreased seed size and weight, starch and cytokinin levels whereas secondary metabolic pathways were significantly enriched. An interesting model that includes the factors responsible for the impact of AtENO2 on the seed size and weight is also presented.

The review by An et al. reports on the current state of the art dealing with embryo-endosperm interaction, the main process controlling grain filling, crucial for yield and quality of the three most important cereals worldwide, rice, wheat, and maize. There are still several open questions regarding the cross-talk and signaling between these two tissues, the role of abscisic acid (ABA) and gibberellin (GA). The review underlines the urgent need for cooperative actions involving geneticists, breeders, physiologists, and agronomists to overcome such constraints.

In line with this claim, the Research Topic also includes two original works focused on seed metabolites in the context of seed vigorization, thus linking fundamental and applied research. Mahmood et al. describe the beneficial effects of He-Ne laser treatments on sunflower seeds, in terms of enhanced drought stress tolerance. Notably, the treatment promoted the accumulation of nutraceutical phenolic compounds and improved the unsaturated-to-saturated fatty acid ratio of the oil as well as carotenoids and total soluble phenolics.

Forti et al. investigated the impact of seed hydropriming on the pre-germinative metabolism in the eggplant crop wild relative *Solanum villosum* Miller (hairy nightshade). Hydropriming followed by dry-back resulted in synchronized germination. The treatments triggered increased tocopherols levels and accumulation of the naturally occurring antioxidant phenolic compounds chlorogenic acid and iso-orientin, found in the dry seeds and *ex novo* accumulation of rutin. The dynamic changes of the pre-germinative metabolism induced by hydropriming are discussed in view of future applications that might boost the use of eggplant CWRs for breeding, upon upgrade mediated by seed technology.

Finally, the role of seed metabolites in pathogen resistance has been investigated by Sistani et al. using an integrative metabolomics-proteomics approach applied to *Pisum sativum* L. in symbiotic association with Arbuscular mycorrhizal fungi (AMF). Seeds of mycorrhizal pea plants were strongly enriched in terms of secondary metabolites with nutritional, medicinal, and pharmaceutical properties, as well as active roles in pathogen response. The reported data show that AMF exert a significant influence on the pea seed metabolome.

## Author Contributions

All authors listed have made a substantial, direct and intellectual contribution to the work, and approved it for publication.

## Conflict of Interest

The authors declare that the research was conducted in the absence of any commercial or financial relationships that could be construed as a potential conflict of interest.

## Publisher's Note

All claims expressed in this article are solely those of the authors and do not necessarily represent those of their affiliated organizations, or those of the publisher, the editors and the reviewers. Any product that may be evaluated in this article, or claim that may be made by its manufacturer, is not guaranteed or endorsed by the publisher.

